# Fine-root carbon and nitrogen concentration of European beech (*Fagus sylvatica* L.) in Italy Prealps: possible implications of coppice conversion to high forest

**DOI:** 10.3389/fpls.2013.00192

**Published:** 2013-06-13

**Authors:** Mattia Terzaghi, Antonio Montagnoli, Antonino Di Iorio, Gabriella S. Scippa, Donato Chiatante

**Affiliations:** ^1^Department of Biotechnology and Life Sciences, University of InsubriaVarese, Italy; ^2^Department of Science and Technology for Environment and Territory, University of MolisePesche, Italy

**Keywords:** fine-root carbon, fine-root nitrogen, stand characteristics, coppice conversion, *Fagus sylvatica* L. fine roots, fine roots soil depth, beech fine roots

## Abstract

Fine-root systems represent a very sensitive plant compartment to environmental changes. Gaining further knowledge about their dynamics would improve soil carbon input understanding. This paper investigates C and N concentrations in fine roots in relation to different stand characteristics resulting from conversion of coppiced forests to high forests. In order to evaluate possible interferences due to different vegetative stages of vegetation, fine-root sampling was repeated six times in each stand during the same 2008 growing season. Fine-root sampling was conducted within three different soil depths (0–10; 10–20; and 20–30 cm). Fine-root traits were measured by means of WinRHIZO software which enable us to separate them into three different diameter classes (0–0.5, 0.5–1.0 and 1.0–2.0 mm). The data collected indicate that N concentration was higher in converted stands than in the coppiced stand whereas C concentration was higher in the coppiced stand than in converted stands. Consequently the fine-root C:N ratio was significantly higher in coppiced than in converted stands and showed an inverse relationship with fine-root turnover rate, confirming a significant change of fine-root status after the conversion of a coppice to high forest.

## Introduction

The Italian National Forest Inventory (SIAN, 2013) indicates that more than 60 percent of Italian forests are maintained under a coppice regime. This situation stems from when there was a high demand for small timber, firewood and charcoal. Now, based on social and economic factors, there is a trend to convert traditional coppice management to high-standard management (Nocentini, 2009). This conversion entails the transition from a condition where a number of stems grow contemporaneously on a single stool to a condition where only one stem is left to continue its growth so that it assumes a larger dimension. At the same time, tree density per hectare is usually decreased.

Forest management practices such as coppice conversion to high forest routinely involve thinning operations. These practices modify stand characteristics (i.e., tree density, canopy cover, stand basal area) and related environmental variables (i.e., soil moisture and temperature, irradiance), leading to changes in the ecophysiological behavior of trees (Aussenac, 2000). In particular, conversion results in considerable alteration of almost all micro-environmental factors that characterize a coppice stand. Various studies show that increase in canopy gap size causes an increase of both seasonal average soil temperatures and soil temperature extremes (Liechty et al., 1992; Hashimoto and Suzuki, 2004). Moreover, seasonal and diurnal differences in maximum-minimum air temperatures increase 15 cm above soil surface as well (Carlson and Groot, 1997).

In the attempt to shed some light on the effects of forest conversion, we studied the effect of conversion of a coppice stand to a high-standard management based on the fine-root component (roots <2 mm in diameter). Our rationale was that fine roots represent the component of a root system that is most sensitive to climate and microclimate variations (Aussenac, 2000; Fotelli et al., 2002), and to stressful conditions such as drought, competition, and herbivory (Lopez et al., 1998; Chiatante et al., 1999, 2005; Glen and Robert, 2006; Withington et al., 2006; Di Iorio et al., 2011; Montagnoli et al., 2012a). Few previous studies showed that stand conversion induces a decrease in the fine-root standing biomass (Lopez et al., 2003; Tufekcioglu et al., 2005). In addition, Fotelli et al. (2002, 2004) reported both an increase and a decrease of fine root biomass in thinned forests, depending on site exposure, whereas Lopez et al. (2003) confirmed that fine-root production is positively affected by management operations. We recently found that the conversion of a beech stand from coppice to high forest may be indirectly responsible, in the fine root component, for a decrease of total biomass and an increase of turnover rate, i.e., a decrease of life-span (Montagnoli et al., 2012b; Table A1). We also found that fine-root biomass production may be transiently stimulated by conversion. Therefore, our earlier findings suggest that the fate of fine roots after conversion is factor that could be considered in the measurement of a forest carbon stock that will be used as an indicator of sustainable forest management (www.fao.org/forestry/ci/en/,2013).

The work reported here builds on previous studies on beech stand conversion. We focus on carbon and nitrogen concentrations in fine roots because these two parameters can be used as indicators of the construction and maintenance costs, respectively, for fine-root biomass (Pregitzer et al., 1997, 2002). In fact, the C concentration of fine roots is associated with construction costs (Gordon and Jackson, 2000; Guo et al., 2004) whereas N concentration is associated with their metabolic activity, respiration and root longevity (Ryan, 1991; Pregitzer et al., 1998; Withington et al., 2006). As a consequence, the C:N ratio can provide an indication of the life-span of fine roots (Withington et al., 2006), i.e., the higher the fine-root C:N ratio, the longer their life-span and the lower the fine-root turnover rate (Pregitzer et al., 2002; McCormack et al., 2012). In particular, we hypothesized that fine-root population in the coppice stand, characterized by a lower fine-root turnover rate than converted stands, might have higher fine-root C:N ratio. This would result in an inverse relationship between fine-root C:N ratio and fine-root turnover rates previously measured by soil coring method.

## Materials and methods

### Site description

The study area was located in the catchments of the Telo stream in the Lombardy Alps (Intelvi Valley, NW Italy, 45° 59′ N, 9° 07′ E) approximately from 1160 to 1200 m above sea level between Lakes Como and Lugano. This area is characterized by a sub-continental climate, with a mean annual precipitation of 1600 mm, occurring in two main periods (April-May and October-November) and a mean annual temperature of 10–11°C. Generally, the area is snow-covered from late October to late March. The 2008 temperatures and precipitations are in accordance with the general trend and magnitude of the past 80 years (weather data from Consorzio dell'Adda, Lombardy, 1920–2000), According to the World Reference Base for Soil Resources (IUSS Working Group WRB, 2006), soil type is Leptosol 40–50 cm deep. Sampling plots were placed in three beech forest stands with different characteristics due to forest management. The three stands were adjacent to each other and located on the same slope (average between 28 and 30°) facing south-west. No significant differences in soil characteristics were recorded between the three stands. Specifically, three beech stands were considered: a residual coppice stand (CpS), the only one left in the area, cut once 40 years ago and then allowed to re-grow from stumps and never recut; two converted stands from coppice to high forest cut in 1994 (CvS 1994) and 2004 (CvS 2004), respectively. Cutting consisted in reducing the number of stems per stool to one per stool, and eliminating exceeding stools thereby reducing stand tree density, and transforming the coppice to high forest. Information from forest inventory indicates similar coppice management practices and stand characteristics for the whole study area. Since beginning of 90s, conversion thinning to high forest fractionated the area in several stands differing in cutting time and tree density. The three selected stands were considered as three different stages in a beech forest successional development with CvS 2004 and CpS representing the younger and older stage, respectively. Therefore, CpS with canopy cover of 94% was considered as the “time zero” before the management change occurred.

### Stand characteristics

Soil temperature was measured during the growing season. Measurements were taken next to the soil cores. On each sampling date, six measurements were made at three soil depths: 5 cm, 15 cm, and 25 cm. Soil temperature was measured using a high accuracy thermometer with a stainless steel probe (mod. CheckTemp 1). The probe utilizes a high-tech NTC thermistor sensor that makes it possible to obtain an extremely high accuracy (± 0.3°C) in a very short time. During the growing season, the mean soil temperature (0–30 cm depth) was lower in the CpS than in both CvSs (Montagnoli et al., 2012b; Table A1).

The tree number and diameter at breast heights (dbh) were surveyed on seven selected 20-m diameter circular-shaped sampling plots per stand (a total of 2199 m^2^ per stand). In order to estimate above-ground biomass, a site specific allometric relationship, which estimates branch and stem biomass from tree dbh, was developed (Montagnoli et al., 2012b). The CpS had higher tree density and above-ground biomass than both CvSs (Table A1). Canopy cover, measured with the hemispherical photo method (Rich, 1990), showed the same trend observed for tree density. In fact, in the CpS, tree canopy almost completely covered the soil surface whereas in the CvS 2004 the cover decreased to half that of the CpS (Table A1). In all three stands the vegetation was dominated by European Beech, while the understory profile was characterized by differences in species and relative abundance between stands. The CpS was characterized by few *Fagus* seedlings, herbaceous species covered 5% of the stand and mosses covered 35%. In the CvS 1994, seedlings of *Fagus* covered up to 15% of the soil surface. Herbaceous species covered from 20 to 50% of the soil and mosses covered only 5% of the soil surface. In the CvS 2004, seedlings of *Fagus* covered up to 15% and seedlings of *Betula pendula* covered 2%. Herbaceous species covered up to 85% of the soil surface and mosses only 1%. More detailed information about stand characteristics were provided in Montagnoli et al. (2012b).

### Fine root measurements

Fine roots were collected at different soil depths using a motor-driven portable root soil core sampler [adapted from Ponder and Alley (1997)] during the 2008 growing season (between May and October). In each stand, four permanent 10-m^2^ plots were set. Two soil cores (4 cm diameter × 30 cm deep) were randomly collected in each plot. Samples were taken when the soil was free of snow cover. Fine roots were sampled on six dates approximately every 30 days for a total of 144 cores (8 cores × 3 stands × 6 collecting dates). The soil cores were separated into three soil layers: 0–10 cm including the humus layer (0–2/3 cm), 10–20 cm and 20–30 cm from the soil surface. Samples were stored in plastic bags at 4°C until processed. Each sample was washed automatically in a filtering nylon bag (300 μm mesh) using a washing machine [adapted from Benjamin and Nielsen (2004)].

Soil-free roots were sorted into color, texture, and shape under a 10× stereomicroscope (Vogt and Persson, 1991). Live fine roots were scanned at resolution of 400 dpi and divided in three subsamples based on three diameter size classes (0–0.5; 0.5–1.0; 1.0–2.0 mm) by using WinRhizo Pro V. 2007d (Regent Instruments Inc., Quebec). Each subsample class was scanned and analyzed in order to obtain the mean class diameter. Subsamples were then oven-dried, weighed and stored in sealed vials to further chemical analysis.

### Fine root carbon and nitrogen concentrations

Fine-root subsamples were ground in liquid N_2_ with mortar and pestle and analyzed for C and N concentrations with a CHN-analyzer (NA-2000 N-Protein; Fisons Instruments S.p.A., Rodano [MI], Italy). The analyzer was calibrated with an atropine standard, and every 10th sample with an atropine sample. The mean total N and C recovery rate for nutrient analysis of atropine was 100.48% (1 *SE* = 0.6%) and 101.02% (1 *SE* = 0.22%), respectively.

### Statistical analysis

Statistical analyses were carried out using the SPSS software package version 12.0 (SPSS Inc, Chicago IL, USA). Fine-root C concentration and fine-root C:N ratio data did not meet the normal distribution and homoscedasticity. A square root transformation produced normal distributions and equal variances. It was not necessary to transform fine-root N concentration data. General linear model (two-way ANCOVA) was performed with forest stand and time as fixed factor and fine-root diameter and soil-depth as covariates. A *post-hoc* multicomparison test (Bonferroni test with a 5% rejection level) was performed on estimated marginal means to detect significant differences between forest stands and sampling times.

## Results and discussion

We measured C and N concentrations in fine roots in three beech forest stands: one maintained as coppice; the other two had been converted to high forest (in 1994 and 2004, respectively) but had a different tree density. All the three stands presented the same soil structural characteristics. Conversion resulted in a decrease in tree density in the CvS 2004, whereas tree density in the CvS 1994 was intermediate between that of CpS and the CvS 2004. The decrease in tree density increased light and soil temperature within the stand (Table A1), but we cannot exclude that the reduced tree density also affected other environmental factors. Given related effects in the fine-root turnover rate due to stand characteristic variations introduced during conversion (Montagnoli et al., 2012b), it is conceivable these changes could correlate to N and C concentrations in fine roots.

### Nitrogen concentration

**Figure 1:**
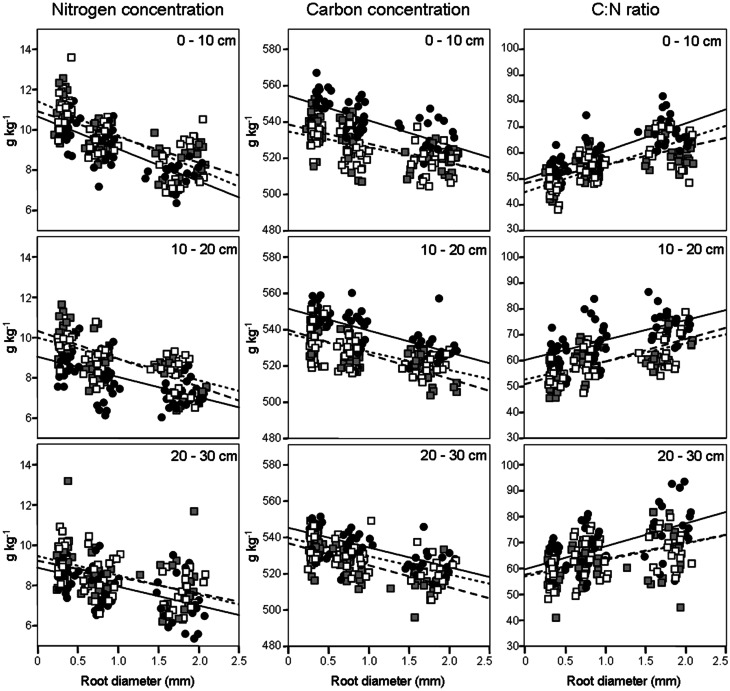
**Fine-root nitrogen and carbon concentrations and C:N ratio (*columns*), according to soil depths (*rows*), in relation to root diameter.** Data refer to 6 sampling dates between 8 May and 17 October 2008. Different dots and regression lines indicate different forest stands (
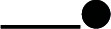
 CpS; 
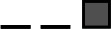
 CvS 1994; 
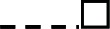
 CvS 2004).

We evaluated N concentrations of fine roots belonging to the three different diameter classes excavated from the three different forest stands at three different soil depths (Figure 1). The N concentration was higher in fine roots with a diameter smaller than 0.5 mm, which live in the most superficial soil layer (0–10 cm). The N concentration in fine roots decreased as root diameter increased in all soil layers considered (Figure 1; Table 1). These findings are in accordance with findings of other similar studies which suggested that N concentration is related to root diameter with the highest concentrations in the thinnest root branches (Gordon and Jackson, 2000; Li et al., 2010) located in the uppermost soil layer (Pregitzer et al., 1998; Persson and Ahlström, 2002; Ayres et al., 2004; Li et al., 2010; Montagnoli et al., 2010). Fine-root diameter showed a significant interaction with soil-depth (*p* = 0.002, Table 1). Indeed, the decrease of N-concentration in relation to soil depth differed depending on fine-root diameter class considered. Very fine roots (<0.5 mm) at 20–30 cm depth showed 16% less N than at 0–10 cm depth, while fine roots with diameter 0.5–1.0-mm and 1.0–2.0-mm showed, respectively, 14 and 7% less N in deeper roots. This result agree with that reported by Pregitzer et al. (1998). The stronger depth-related variation of very fine roots (<0.5 mm) will remain to deepen and could be related to their uptake function, which means that very fine roots are more sensitive to changes in soil features than larger roots. We have limited our investigation to fine roots with a maximum diameter of 2 mm and have divided them into diameter classes. Although we do not know whether functional differences exist between these three diameter classes, we cannot exclude that, also in our case, fine roots with a diameter smaller than 0.5 mm could play a role in N-uptake function as suggested by Guo et al. (2008) and Hishi (2007). If this is the case, we could speculate that fine roots belonging to the two larger diameter classes might play a role in transport and storage function (Hishi, 2007; Guo et al., 2008).

**Table 1:** **General linear model analysis (two-way ANCOVA) for the effects of forest stand and time on fine-root N and C concentrations and C:N ratio**.

**Parameter**	***F***	***p*-value**
**NITROGEN CONCENTRATION**		
Fine-root diameter (c)	69.138	<0.001
soil-depth (c)	60.160	<0.001
forest stand	19.794	<0.001
time	7.850	<0.001
Fine-root diameter (c) × soil-depth (c)	9.457	0.002
**CARBON CONCENTRATION**		
Fine-root diameter (c)	235.575	<0.001
soil-depth (c)	2.509	0.114
forest stand	67.113	<0.001
time	11.143	<0.001
**C:N RATIO**		
Fine-root diameter (c)	152.441	<0.001
soil-depth (c)	74.921	<0.001
forest stand	17.693	<0.001
time	10.095	<0.001

Fine-root diameter and soil-depth were treated as covariates (c). Non-significant interactive effects were excluded from the model.

Regarding forest stands, we found significant lower N values in CpS than both CvSs (Bonferroni test, *p* < 0.001), mainly at a depth of 10–20 cm (11% less N, Figure 1). Higher N concentrations in both CvSs could be related to the higher soil temperatures, as found in other studies (Geßler et al., 1998; Fotelli et al., 2002, 2004; Nahm et al., 2006).

Previous studies showed that the N concentration in fine roots is directly related to their metabolic activity and respiration, and inversely to their longevity (Ryan, 1991; Pregitzer et al., 1998; Withington et al., 2006; McCormack et al., 2012). Therefore, our finding that N concentration was significantly higher in the two CvSs than in the CpS suggests that variations introduced by conversion may be responsible for the increased metabolic activities of fine roots, which in turn, would lead to an increase of their growth rate (Valverde-Barrantes et al., 2007) and a shorting of their life-span. This hypothesis is consistent with our previous findings (Montagnoli et al., 2012b) that the turnover rate of fine roots increases, and consequently the life-span decreases, as a result of change in stand characteristics due to conversion operations.

In our experiment, variations in the fine-root N concentration were similar in the three beech forest stands during the vegetative season (from May to October, Table 2). In all three stands, the N concentration significantly decreased during spring, and returned to similar values at the end of the growing season. This pattern of N concentration variation is in line with the report that temperate forests are characterized by seasonal variations of N concentration (Cerasoli et al., 2004; Nahm et al., 2006). Therefore, in analogy with Millard (1989) and Fotelli et al. (2002), we suggest that also in our beech forest stands the decrease in N could be associated to utilization of the N reserve in order to support newly developing tissues and the increase with restoration of the N-depleted reserves.

**Table 2:** **Estimated marginal mean values (means adjusted for soil-depth and diameter class covariates, *N* = 36) of fine-root nitrogen and carbon concentration and C:N ratio**.

	**Nitrogen concentration (g kg^−1^)**			**Carbon concentration (g kg^−1^)**			**C:N ratio**		
**Sampling date**	**CpS**	**CvS 1994**	**CvS 2004**	**CpS**	**CvS 1994**	**CvS 2004**	**CpS**	**CvS 1994**	**CvS 2004**
8 May	8.49ab x	9.36a y	9.29a y	536.5a x	521.8a y	528.9ab y	62.8ab x	58.7ab x	57.9a x
20 June	7.69b x	8.95ab y	8.60b y	535.6a x	526.4ab y	523.9a y	71.0c x	62.7b y	59.9a y
12 July	8.15ab x	8.37a x	8.75ab x	536.6a x	533.5c xy	529.1ab y	67.4bc x	65.2b x	65.8b x
26 August	8.32ab x	8.79a x	8.77ab x	544.9b x	529.9bc y	534.3b y	66.8abc x	62.7b x	58.2a x
24 September	8.63a x	8.94ab x	9.18ab x	541.7ab x	528.3abc y	529.8ab y	63.5ab x	59.9ab x	60.4ab x
17 October	8.82a x	9.62a y	9.72a y	534.0a x	521.3a y	523.6a y	60.0a x	55.8a x	59.3a x

a, b, and c indicate significant differences between sampling dates. For each sampling date x and y indicate significant differences between stands (Bonferroni test, p < 0.05).

### Carbon concentration

Previous studies identified considerable differences in C concentration in the fine roots of different species, and showed that C concentration is related positively to root diameter (Gordon and Jackson, 2000; Pregitzer et al., 2002). In contrast, another study reported that C concentration was highest in roots with the thinnest diameter (Goldfarb et al., 1990). We found that C concentration did not differ among soil depths (*p* = 0.144) and significantly decreased with increasing root diameter (Table 1; Figure 1). The same result was found by Gaul et al. (2009) in a Norway spruce forest. Moreover, fine-root C concentration was significantly higher in the CpS than in both CvSs (Bonferroni test, *p* < 0.001) highlighting a higher investment of carbon in CpS than CvSs fine roots, although differences are small (1.5–3% of variation, Figure 1). Our findings could be related to a higher content of secondary metabolites (i.e., lignin and tannins, Harborne, 1980). In fact, secondary metabolites have a C content higher than compounds like cellulose and other sugars (Chua and Wayman, 1979; Krässig, 1993), therefore an increase in secondary metabolites would result in an increase in total C concentrations. Alternatively, we cannot exclude that a higher C concentration could derive from a lower cellulose and/or total-non-structural carbohydrate (TNC) content (Nguyen et al., 1990; Guo et al., 2004), e.g., less secondary xylem and/or lower starch concentration in CpS fine roots, although this still need further investigation.

In regard to the seasonal variation, Goldfarb et al. (1990) suggested that C concentration in fine roots is higher in early summer than in spring or autumn. We confirm the significant variation of C concentration in fine roots during the year with a peak in July or August depending upon the forest stand (Table 2). The peak of C concentration found by us during summer could be related to the maximum vegetative activity which requires a reduction of investment in TNC. This possibility is in accordance with data of Cerasoli et al. (2004) who reported, during the growing season, the highest C concentration in roots while TNC levels were the lowest. The rapid decrease of C concentration following the peak could be related to the end of the growing season and therefore to the need to restore the sugar reserve (Nguyen et al., 1990).

### Fine-root C:N ratio

Fine-root C:N ratio data in the present study ranged from 39.6 to 93.4 (Figure 1) and were of the same magnitude as other published values for the same tree species (Persson and Ahlström, 2002; Ayres et al., 2004; Zang et al., 2011). Moreover, we found an increase of fine-root C:N ratio with depth that was related to the depth-dependent pattern of N concentration (Figure 1). For the same reason, the fine-root C:N ratio significantly increased during spring, and returned to similar values at the end of the growing season (Table 2).

**Figure 2:**
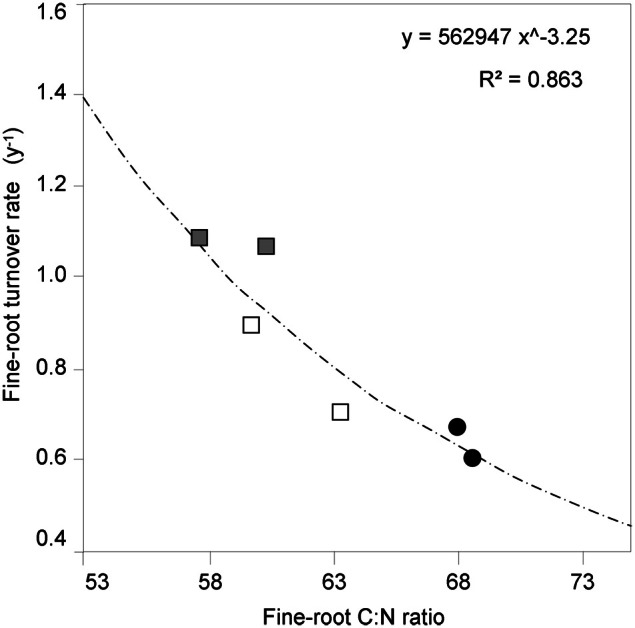
**The relationship between fine-root C:N ratio and fine-root turnover rate.** C:N ratio data are estimated marginal mean values (means adjusted for diameter class covariates, *N* = 72). Significant turnover rate data (*p* < 0.05) are from Table 3 in Montagnoli et al. (2012b) and refer to two different soil depths. Different symbols indicate different forest stands (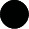 CpS; 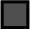 CvS 1994; 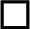 CvS 2004).

It is known (as mentioned above) that fine-root C:N ratio has a direct relation with fine-root life-span (Pregitzer et al., 2002; Tjoelker et al., 2005; Withington et al., 2006; Gaul et al., 2009; McCormack et al., 2012). It can also cast light on the relationship between costs for fine-root biomass construction (in term of C concentration) and costs for biomass maintenance (in terms of N concentration) (Pregitzer et al., 1997, 2002). In accordance with our hypothesis, the fine-root C:N ratio differed significantly between forest stands (*p* < 0.001, Table 1) and was significantly higher in the CpS than in both CvSs (Bonferroni test, *p* < 0.001), independently from soil depth (Figure 1; Table A2). Moreover, fine-root C:N ratio was inversely related to fine-root turnover rate [data from Montagnoli et al. (2012b)] with a significant inverse power regression (*R*^2^ = 0.863; *p* = 0.007; Figure 2). In particular, these results highlight that in terms of cost-benefit ratio, the higher C investment in the construction of CpS fine roots is balanced by lower maintenance cost (lower N concentration) and by higher lifespan.

In conclusion, our study shows that changing in stand characteristics due to conversion operations (from coppice to high forest) affects C and N concentrations of fine roots. Converted stands showed lower fine-root C concentration and higher fine-root N concentration than coppice. Consequently the fine-root C:N ratio was significantly higher in coppiced than in converted stands. These results support our previous finding (Montagnoli et al., 2012b) that a coppiced forest left to grow for 40 years is characterized by fine roots with a longer life-span than those living in stands recently converted to high forest. Therefore, in our coppiced forest, fine-root carbon stock lasted longer than converted. Moreover, the thinnest root component (<0.5 mm) appears to be more sensitive to changes in stand characteristics than other root diameter classes.

### Conflict of interest statement

The authors declare that the research was conducted in the absence of any commercial or financial relationships that could be construed as a potential conflict of interest.

## Appendix

**Table A1:** **Beech above-ground characteristics, live fine-root biomass and soil temperature of the three investigated stands**.

**Forest stand**	**Density (trees ha^−1^)**	**Canopy cover (%)^a^**	**Above-ground biomass (Mg ha^−1^)^b^**	**Live fine-root biomass (g m^−2^)^c^**	**Soil temperature (°C)^d^**
CpS	724 ± 35	94.2 ±0.6	248.5 ± 15.6	230.0 ± 17.2	10.24 ± 0.30
CvS 1994	279 ± 24	74.2 ± 5.5	123.7 ± 7.3	144.8 ± 14.7	11.26 ± 0.32
CvS 2004	167 ± 20	54.3 ± 3.2	91.8 ± 20.2	119.4 ± 13.7	12.23 ± 0.36

Data shown are means ±S.E. dbh, diameter at breast height.

a Canopy cover values are the mean of 10 replicates.

b Above-ground biomass values are the mean of seven replicates.

c Fine-root biomass values are the mean of 32 samples (eight sampling time X four plots).

d Soil temperature (0–30 cm) is referred to the mean of three soil depths (5, 15, and 25 cm) and each value is the mean of four replicates for eight sampling dates (May 2008–April 2009). Data are from Montagnoli et al. (2012b).

**Table A2:** **Fine-root nitrogen and carbon concentrations and C:N ratio of three diameter classes**.

	**Diameter class 0–0.5 mm**			**Diameter class 0.5–1 mm**			**Diameter class 1–2 mm**		
**Soil depth (cm)**	**CpS**	**CvS 1994**	**CvS 2004**	**CpS**	**CvS 1994**	**CvS 2004**	**CpS**	**CvS 1994**	**CvS 2004**
**NITROGEN**	**(g kg^−1^)**			**(g kg^−1^)**			**(g kg^−1^)**		
0–10	10.3 ± 0.3	10.6 ± 0.3	11.2 ± 0.3	9.7 ± 0.4	9.0 ± 0.3	9.4 ± 0.3	8.2 ± 0.4	8.4 ± 0.3	8.1 ± 0.3
10–20	8.9 ± 0.2	10.2 ± 0.3	9.8 ± 0.1	8.0 ± 0.3	8.8 ± 0.2	8.9 ± 0.3	7.2 ± 0.2	7.9 ± 0.3	8.2 ± 0.2
20–30	8.6 ± 0.2	9.3 ± 0.4	9.5 ± 0.2	8.1 ± 0.3	7.9 ± 0.2	8.3 ± 0.3	7.3 ± 0.4	7.7 ± 0.5	7.8 ± 0.2
**CARBON**	**(g kg^−1^)**			**(g kg^−1^)**			**(g kg^−1^)**		
0–10	551.4 ± 2.3	536.8 ± 2.6	533.3 ± 1.9	540.4 ± 2.0	528.3 ± 3.1	525.2 ± 2.3	527.3 ± 3.3	519.4 ± 1.8	519.7 ± 2.6
10–20	548.8 ± 1.4	536.6 ± 2.6	536.8 ± 3.7	538.8 ± 3.5	526.6 ± 2.0	528.2 ± 2.1	530.1 ± 3.0	518.0 ± 2.6	521.9 ± 1.8
20–30	541.7 ± 2.5	534.5 ± 2.0	538.8 ± 3.3	536.2 ± 2.2	525.7 ± 2.0	530.9 ± 2.4	525.9 ± 2.3	515.0 ± 2.7	521.4 ± 2.4
**C:N RATIO**									
0–10	53.8 ± 1.4	51.0 ± 1.2	47.8 ± 1.1	57.1 ± 2.4	59.4 ± 2.3	56.3 ± 1.7	66.0 ± 2.6	62.5 ± 2.0	65.5 ± 2.3
10–20	62.3 ± 1.5	53.1 ± 1.3	54.8 ± 0.9	68.5 ± 2.4	60.2 ± 1.5	59.8 ± 1.6	73.9 ± 1.7	66.9 ± 2.3	64.1 ± 2.1
20–30	63.6 ± 1.4	58.1 ± 2.0	57.3 ± 1.5	67.6 ± 2.5	67.0 ± 1.5	64.7 ± 2.2	74.6 ± 3.6	69.6 ± 3.4	67.7 ± 2.0

Values refer to three soil depths each covering 10 cm and three different forest management stands. Each value represents a mean of 24 samples ± S.E.
